# Stress-Assisted Amorphization and Dislocation-Mediated Plasticity in Silicon near Its Melting Point: A Molecular Dynamics Study

**DOI:** 10.3390/ma19184014

**Published:** 2026-09-21

**Authors:** Zhangyong Chang, Yuxia Zhang, Zhigang Xiao, Ming Cheng, Zhenhua Chen, Cuiling Hou

**Affiliations:** Jiangxi Province Key Laboratory of Microstructure Function Materials, School of Science, Jiujiang University, Jiujiang 332005, China

**Keywords:** silicon, amorphization, molecular dynamics, mechanical properties, dislocations

## Abstract

**Highlights:**

• MD simulations reveal a two-stage elastoplastic transition in silicon near its melting point.
• Compressive stress induces uniform amorphization throughout the crystal before yielding.
• Shockley partial dislocations nucleate on {111} planes at the upper yield strength.
• Amorphous phase recrystallizes during stress relaxation as dislocations initiate plastic flow.
• Stress-assisted amorphization and dislocation plasticity occur sequentially, not competitively.

**Abstract:**

Whether the plasticity of silicon at high temperatures originates from dislocation slip or solid-state phase transition remains difficult to investigate directly through experimental observation due to its microscopic nature. Using molecular dynamics simulations, we investigated the deformation behavior of silicon crystals along four typical crystallographic directions at a reduced temperature of T/T_m_ = 0.88 (T_m_ denotes the melting point of silicon predicted by the Tersoff potential). The results indicate that prior to yielding, compressive stress induces uniform amorphization throughout the crystal. Once the critical stress is exceeded, Shockley partial dislocations nucleate on the {111} crystal planes, initiating plastic flow, while the amorphous phase undergoes recrystallization during stress relaxation. These findings demonstrate that at high temperatures, the plasticity of silicon involves both stress-assisted amorphization and dislocation-mediated plasticity, which occur sequentially rather than competing with one another. This study provides an atomic-scale rationale that consolidates the long-standing process guidance for suppressing dislocation defects in the directional solidification growth of multicrystalline silicon.

## 1. Introduction

Multicrystalline silicon (mc-Si) solar cells combine relatively low production cost with competitive conversion efficiency, and remain important for cost-sensitive photovoltaic applications, although monocrystalline wafers now dominate the photovoltaic market. The efficiency deficit of mc-Si solar cells stems primarily from crystal defects, such as grain boundaries, twins, dislocations, and impurities, introduced during casting. Among these, dislocations are the most detrimental to device performance [1,2,3,4,5]. Because dislocations multiply most readily while the ingot is still hot, understanding how silicon deforms near its melting point is essential for inhibiting dislocation generation during directional solidification. Silicon also offers unusually well-controlled solidification and growth conditions, which have made it a model material for fundamental studies of mechanical behavior. Experimental work dating back to the 1960s has documented its high-temperature ductility and low-temperature brittleness [6,7,8,9], and an extensive theoretical literature has clarified the dislocation types and core configurations in the diamond lattice [10,11,12,13,14]. For bulk crystals deformed above the brittle-to-ductile transition temperature (about 700 K under conventional quasistatic testing, although the exact value depends strongly on strain rate, sample geometry, and surface condition), the plastic mechanisms are relatively well established: plasticity in this regime is mainly mediated by partial dislocations gliding on the {111} planes of the diamond cubic structure [15,16]. What remains debated is the mechanism by which the crystal transitions from elastic to plastic response, specifically whether high-temperature plasticity is governed by dislocation activity or phase transformation. Resolving this question experimentally is extremely challenging, because the relevant microscopic events cannot be observed directly at these temperatures. Molecular dynamics (MD) simulation is one of the few viable methods that can track atomic trajectories on picosecond timescales at such temperatures, enabling real-time observation of phase transitions, as well as dislocation nucleation and evolution. MD has therefore become a standard tool for probing the elastic and plastic behavior of silicon crystals [9,17,18,19]. The present study addresses whether the elastoplastic transition of silicon near the melting point is governed by stress-assisted amorphization or dislocation-mediated plasticity, and explores the interaction mechanism of these two processes at the atomic scale. The findings obtained in our work resolve this long-standing debate concerning the high-temperature plasticity of silicon, and consolidate the long-standing process guidance for suppressing dislocation defects in directionally solidified silicon ingots.

## 2. Model and Method

### 2.1. Silicon Crystal Model

**Figure 1:**
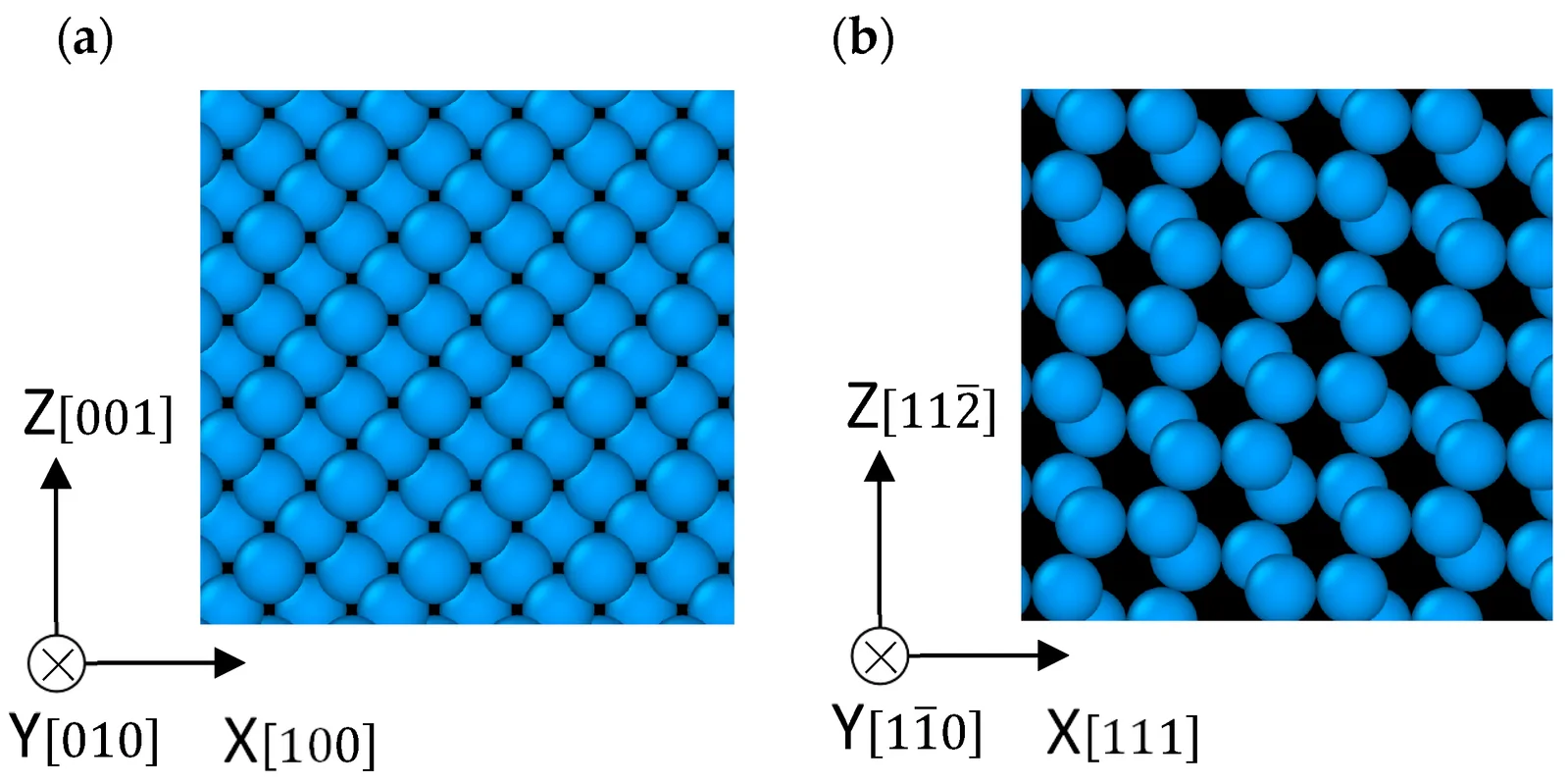
Molecular dynamics models of silicon crystals used in the tensile and compressive deformation simulations: (**a**) Model A; (**b**) Model B (blue spheres denote atoms on the diamond cubic lattice).

The two molecular dynamics models used for the high-temperature deformation simulations are shown in Figure 1. They are referred to as Model A and Model B and are used to investigate the orientation dependence of the mechanical response of crystalline silicon. In Model A, the X, Y, and Z axes of the simulation cell lie along the $\left[100\right]$, $\left[010\right]$, and $\left[001\right]$ directions, respectively; in Model B, they lie along the $\left[111\right]$, $\;\left[1\bar{1}0\right]$, and $\left[11\bar{2}\right]$ directions, respectively. Together, the two cells provide the four most commonly studied loading orientations of silicon. Model A comprises 18 × 18 × 18 unit cells(8 atoms each), corresponding to an initial box of 97.7 × 97.7 × 97.7 Å^3^, and Model B comprises 18 × 18 × 18 unit cells (12 atoms each), corresponding to an initial box of 169.3 × 69.1 × 119.7 Å^3^. The two models contain 46,656 and 69,984 atoms, respectively. These system sizes are comparable to those employed in previous MD studies of silicon plasticity [18,19]. To assess possible finite-size effects, a control simulation with the cell size doubled along the loading direction was performed for [100] compression: the same sequence of amorphization followed by dislocation nucleation was observed, and the critical strain and upper yield strength changed by less than 0.5%, indicating that finite-size effects do not alter the conclusions of this work. In both cells, the atoms are initially placed on a diamond cubic lattice with a lattice parameter of a_0_ = 5.43 Å. This value is the equilibrium lattice parameter of the Tersoff potential at low temperature and coincides with the experimental room-temperature value; the thermal expansion at T/T_m_ = 0.88 is accommodated during the NPT equilibration described below. Periodic boundary conditions are imposed in all three directions to mimic bulk silicon.

### 2.2. Simulation Method

The interatomic interactions were described by the Tersoff Si potential [20], which has been extensively validated for silicon and reproduces both the elastic and plastic properties of this material with reasonable accuracy [21,22,23,24]. One recognized shortcoming of this potential is its overestimation of the melting point of silicon (about 2480 K in simulation, compared with 1687 K experimentally). All results are therefore reported in terms of the reduced temperature T/T_m_, which allows direct comparison with experiments and with simulations using other interatomic potentials. Before loading, each crystal was equilibrated for 1 ns at T/T_m_ = 0.88, yielding a thermally stable initial configuration; during this equilibration, the simulation box was allowed to expand freely in all three directions, and the equilibrated lattice parameter at T/T_m_ = 0.88 was 5.49 Å (box dimensions immediately before deformation: 98.9 × 98.9 × 98.9 Å^3^ for Model A and 171.2 × 69.9 × 121.0 Å^3^ for Model B). Uniaxial tension and compression were then simulated: in Model A, the load was applied along the $\left[100\right]$ direction, and in Model B along the $\left[111\right]$, $\left[1\bar{1}0\right]$, and $\left[11\bar{2}\right]$ directions. The uniaxial strain was ramped continuously at a fixed rate of 1.0 × 10^8^ s^−1^. This rate exceeds laboratory deformation rates by orders of magnitude, but it lies at the lower end of the range accessible to MD and is necessary to resolve microscopic deformation mechanisms within the available time window. All simulations were performed in the isothermal-isobaric (NPT) ensemble. During deformation, the box length along the loading direction was varied at the imposed strain rate using the *fix deform* command, while the two transverse directions were coupled to a Nosé-Hoover barostat at zero external pressure (1 atm) so that the lateral stresses could relax; the temperature was maintained at T/T_m_ = 0.88 using a Nosé-Hoover thermostat. The reported stress is the component of the virial stress tensor along the loading direction, averaged over all atoms. Each loading condition was repeated five times with independent initial configurations generated from different random velocity seeds; the critical strain, the upper yield strength, and the peak amorphous fraction obtained from these runs showed relative standard deviations below 3%, confirming the numerical reliability of our protocol.

All simulations were carried out with the Large-scale Atomic/Molecular Massively Parallel Simulator (LAMMPS) [25]. Each structure was energy-minimized prior to loading to eliminate residual stress. Crystalline and amorphous atoms, stacking faults, twin boundaries, and dislocations were subsequently identified using OVITO [26]. The Identify Diamond Structure and Elastic Strain Calculation modifiers were used for structural classification, and dislocation lines were extracted and indexed with the Dislocation Extraction Algorithm (DXA) [27]. In this work, an atom is classified as amorphous when the Identify Diamond Structure modifier assigns it to neither the cubic diamond nor the hexagonal diamond (stacking-fault) environment; atoms belonging to dislocation cores identified by DXA are excluded from the amorphous population wherever possible. To verify that this classifier does not spuriously label a highly strained crystal as amorphous, a control simulation was performed in which the equilibrated crystal was subjected to homogeneous hydrostatic compression up to 12 GPa at T/T_m_ = 0.88: the amorphous fraction remained below 0.02%, confirming that the criterion correctly retains the crystalline assignment under purely elastic distortion. We nevertheless note that structural classifiers cannot fully exclude transient thermal disorder or highly deformed crystal environments, and the amorphous fractions reported below should therefore be regarded as upper-bound estimates with an uncertainty of a few percent.

We further note that reduced-temperature normalization does not guarantee that defect energetics, stacking-fault energies, amorphization behavior, and dislocation mobility scale identically for different interatomic potentials. The Tersoff potential is known to underestimate the stacking-fault energy of silicon, whereas improved Stillinger-Weber parametrizations provide a better description of defects and plasticity [28]. As a cross-check, the [100] compression was repeated with the Stillinger-Weber potential of Pizzagalli et al. [28]: the same qualitative sequence—amorphization before yielding, followed by the nucleation of Shockley partial dislocations—was reproduced, although the critical stresses differed by 3%. The mechanistic conclusions of this work are therefore expected to be robust with respect to the choice of potential, whereas the quantitative values of the critical stresses and amorphous fractions should be regarded as potential-dependent.

## 3. Results and Discussion

**Figure 2:**
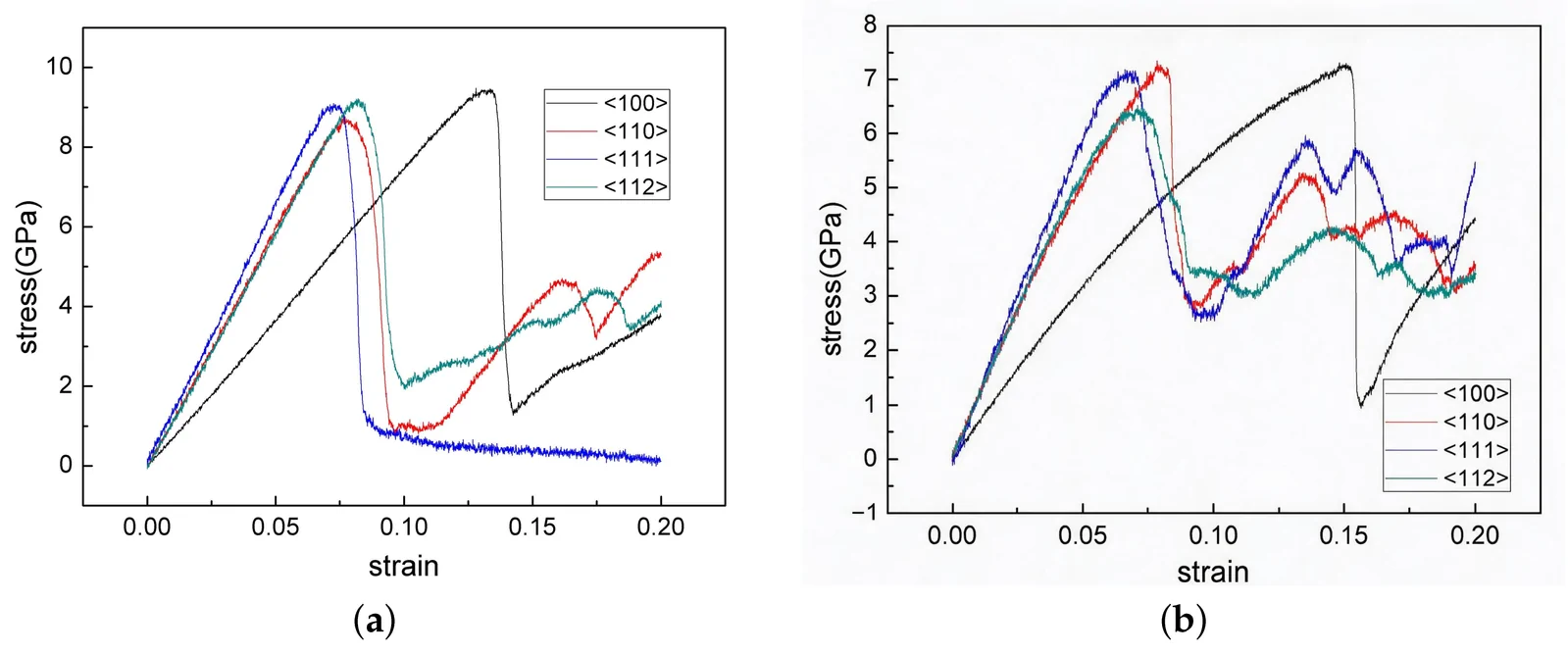
Stress–strain curves of silicon crystals under tensile and compressive deformation: (**a**) tension; (**b**) compression.

**Figure 3:**
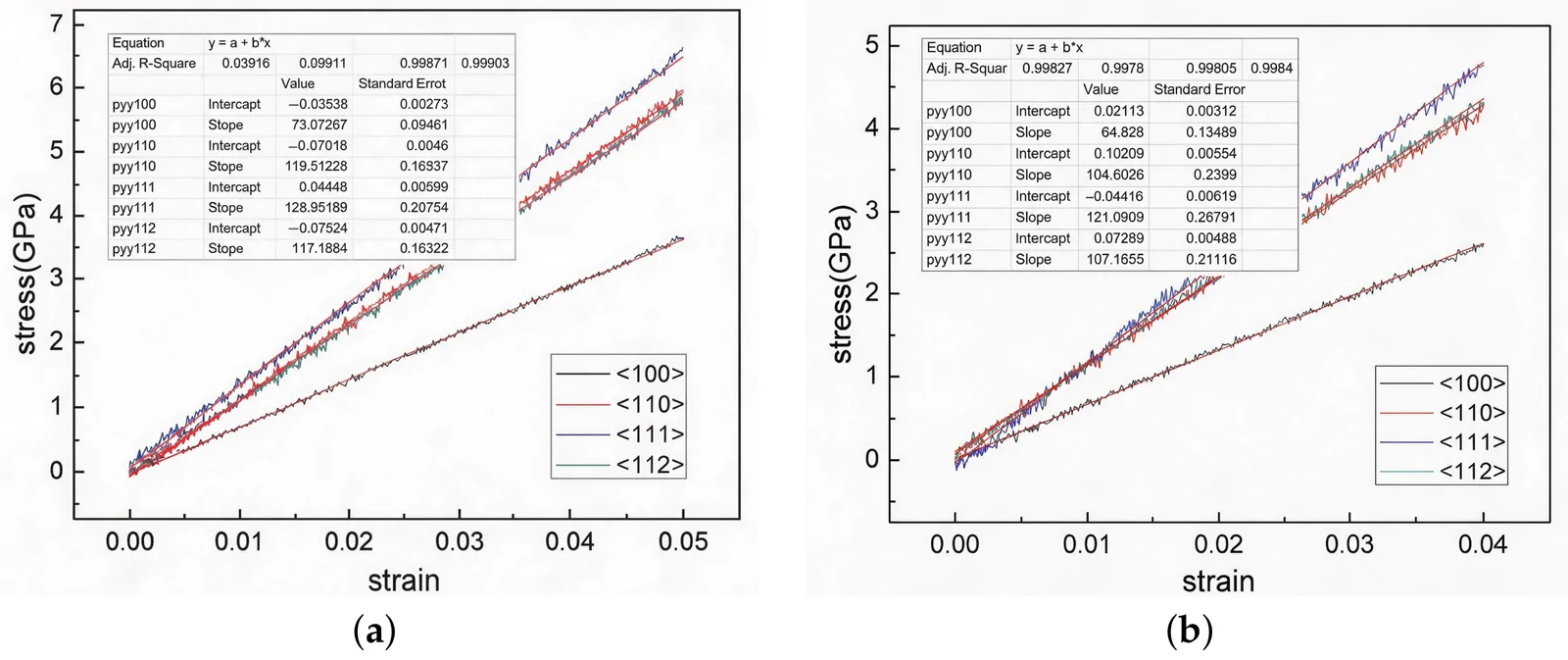
Linear fits to the elastic portions of the stress–strain curves: (**a**) tension; (**b**) compression.

Figure 2a,b present the stress–strain curves obtained during tension and compression along the four representative crystallographic directions ($\left[100\right]$, $\;\left[1\bar{1}0\right]$, $\;\left[111\right]$, $\;\left[11\bar{2}\right]$) at the reduced temperature T/T_m_ = 0.88. In tension, all four curves rise almost linearly up to the maximum stress, exhibiting a distinct linear elastic regime that persists even at temperatures approaching the melting point. In compression, however, the curves remain linear only up to a strain of about 0.04, indicating a significantly narrower elastic range across all four orientations. Figure 3a,b show the linear fits to the elastic portions of the curves, and their slopes provide the tensile and compressive Young’s moduli for the four orientations, which are collected in Table 1. The linear fits were restricted to the strictly elastic initial portions of the curves (strain intervals of 0–0.05 in tension and 0–0.04 in compression), so that the fitted slopes represent the initial tangent (Young’s) moduli; the tension-compression asymmetry developing at larger strains is discussed below as a nonlinear mechanical response. At larger strains, the response in Figure 2 becomes strongly nonlinear, most visibly in compression, until a critical stress is reached; the stress then falls abruptly and plastic flow begins. We refer to the peak stress at the end of this nonlinear stage as the upper yield strength, and the corresponding values for tension and compression along the four directions are likewise given in Table 1.

**Figure 4:**
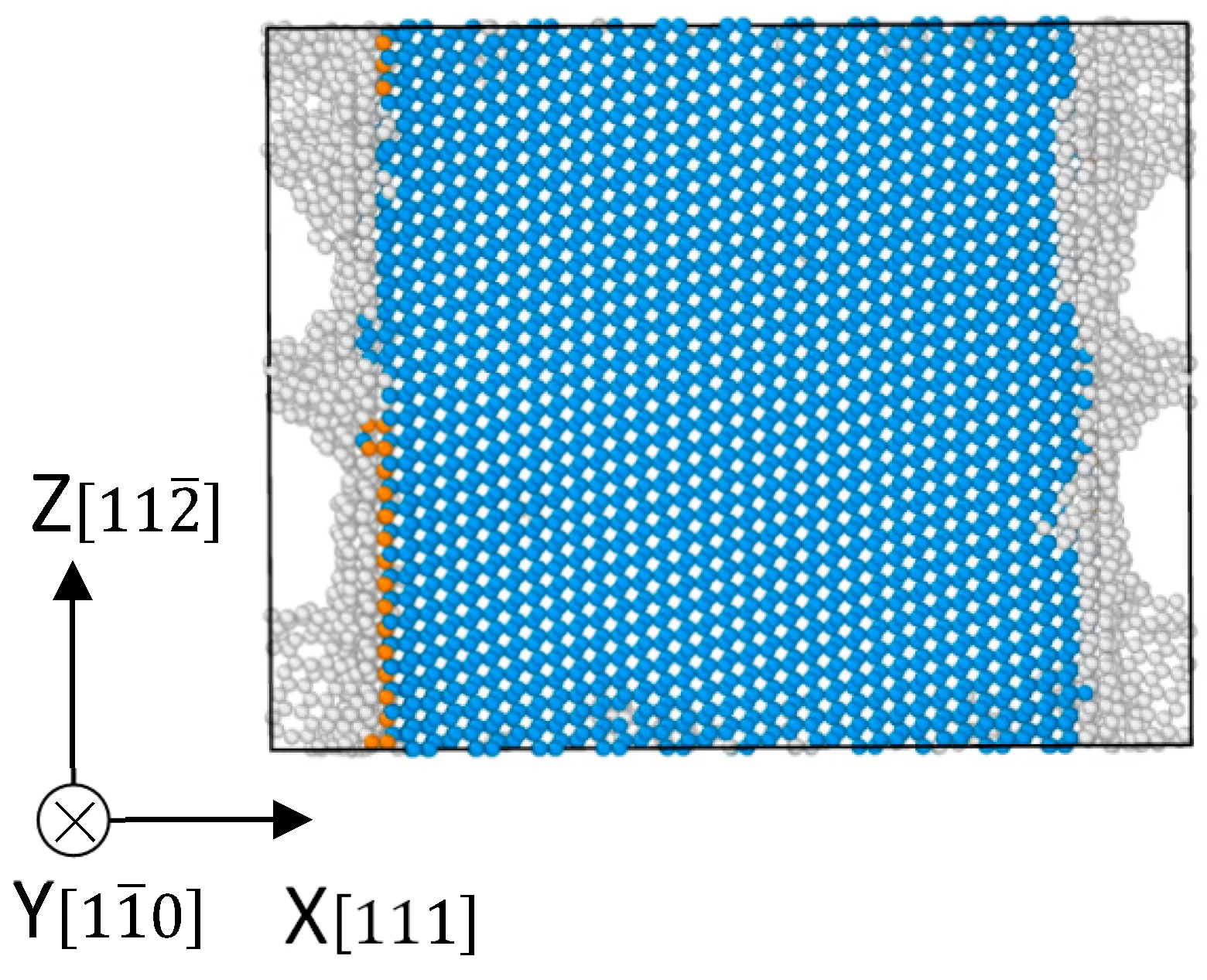
Snapshot of brittle fracture along the (111) plane of a silicon crystal during tensile deformation along the [111] direction at high temperature (blue spheres denote diamond cubic atoms; gray spheres and orange spheres denote defect or amorphous atoms). The snapshot was taken at a tensile strain of 0.07.

As shown in Figure 2a, at T/T_m_ = 0.88, the tensile stress–strain curve for loading along the $\left[111\right]$ direction differs from the other three: after the peak stress, the crystal does not enter a plastic stage but fails directly by brittle fracture along the $\left(111\right)$ plane, and a snapshot of the resulting fracture on the $\left(111\right)$ plane is displayed in Figure 4. Even at this temperature, tensile loading along the $\left[111\right]$ direction still results in brittle fracture rather than thermally assisted plastic flow. This behavior is structural in origin: the {111} planes are the close-packed planes of silicon, and the interplanar bonding across them is relatively weak. In Figure 4, the fracture is identified by the opening of a sharp crack that separates the crystal along the (111) plane; the thin amorphous layer decorating the fracture surfaces forms locally during crack opening and the associated stress release, while the bulk of the crystal remains in the diamond cubic structure and shows no dislocation activity, which distinguishes this event from plastic flow.

**Figure 5:**
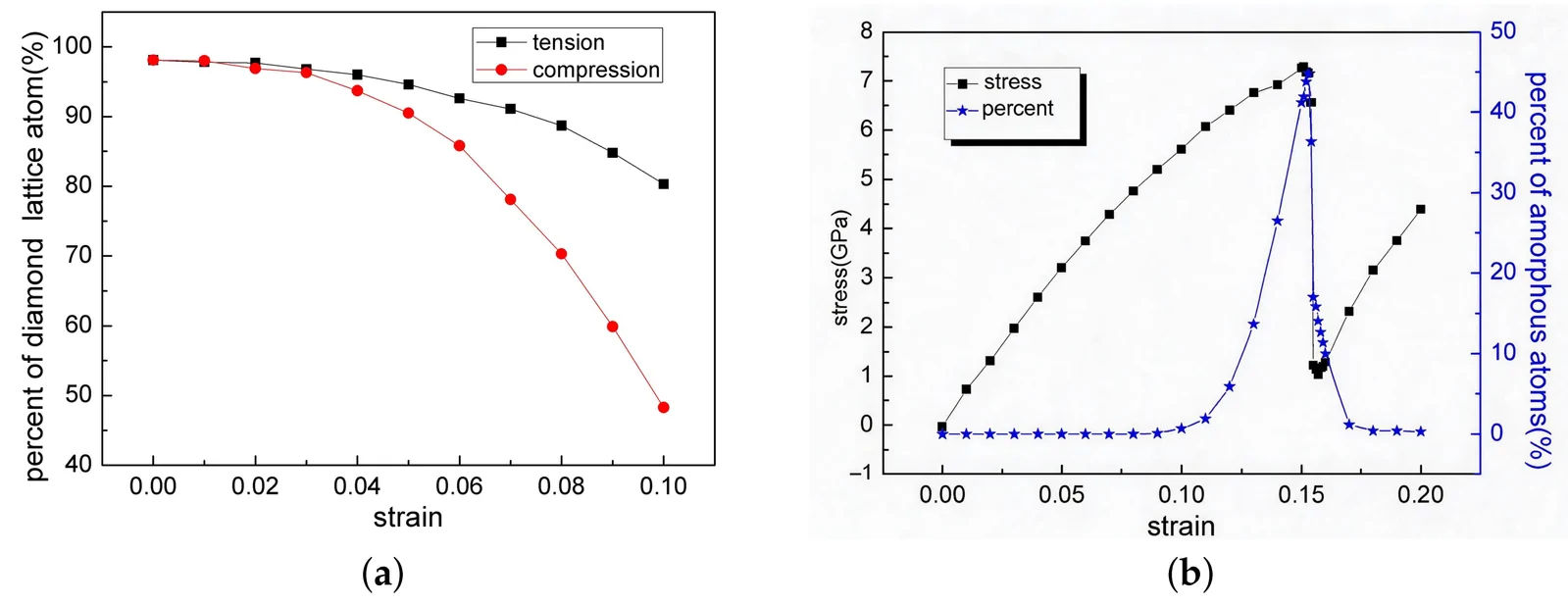
(**a**) Fraction of atoms retaining the diamond cubic lattice as a function of strain during deformation along the $\left[100\right]$ direction of the Si crystal; (**b**) evolution of the amorphous fraction and the stress during compression along the $\left[100\right]$ direction.

Figure 5a plots the fraction of atoms that retain the diamond cubic lattice as a function of strain for tension and compression along the $\left[100\right]$ direction. Over the whole strain range, this fraction is lower in compression than in tension. It should be noted that the non-diamond population in Figure 5a includes all atoms not assigned to the cubic diamond environment—amorphous atoms, hexagonal-diamond (stacking-fault) atoms, dislocation cores, and other defect atoms—whereas the amorphous fraction in Figure 5b counts only atoms with a disordered, non-crystalline environment (see Section 2.2). The two quantities are therefore not complementary: at a strain of 0.10, most non-diamond atoms are locally distorted crystalline and defect atoms rather than truly amorphous ones, which reconciles the reduction in the diamond fraction to below 50% in Figure 5a with the amorphous fraction of less than 5% in Figure 5b. The same compression-induced reduction in the fraction of diamond cubic atoms observed along the [100] direction is also seen for the other three orientations ($\left[1\bar{1}0\right]$, $\;\left[111\right]$, $\;\left[11\bar{2}\right]$), and it accounts for the progressive softening of the compressive stress–strain response at high temperature. Elastic strain is accommodated by the stretching and contraction of interatomic bonds; when fewer silicon atoms remain in the diamond cubic lattice structure, the Young’s modulus decreases as a result. Figure 5a further shows that under compression, this fraction decreases progressively more rapidly as strain increases, a trend that matches the behavior in Figure 2, where the slope of the $\left[100\right]$ compressive stress–strain curve decreases markedly with strain. Compression therefore accelerates the rupture of Si-Si bonds in the diamond cubic structure, leading to progressive amorphization, and the elastic stiffness of the crystal decreases as well.

During directional solidification of mc-Si, compressive stresses can develop between adjacent grains near the solid–liquid interface, which arise from radial thermal gradients at the growth front and volumetric expansion during solidification. Under compressive loading, the strain in the silicon crystal increases with applied stress, and the diamond cubic lattice becomes progressively more distorted. At high temperature, strong thermal vibrations of the silicon atoms assist the partial breaking of the saturated, directional Si-Si bonds in the distorted lattice, and the affected region ultimately transforms from the crystalline to the amorphous state. Amorphization is thus driven mainly by mechanical loading at elevated temperatures, while thermal vibrations facilitate bond breakage. Figure 5b tracks the stress and the amorphous fraction as functions of strain during compression along the $\left[100\right]$ direction. Once the stress reaches the critical value $\sigma_{c}=5.24\;GPa$, the crystalline-to-amorphous transition begins. The amorphous fraction then grows rapidly until the stress reaches the upper yield strength $\sigma_{U}=7.23\;GPa$, at which point the amorphous fraction peaks at 44.8%. The critical amorphization stress σ_c_ was also determined for compression along the other three orientations, yielding 5.85, 6.45, and 6.26 GPa for the $\left[1\bar{1}0\right]$, $\left[111\right]$, and $\left[11\bar{2}\right]$ directions, respectively; stress-assisted amorphization thus precedes yielding for all orientations studied.

**Figure 6:**
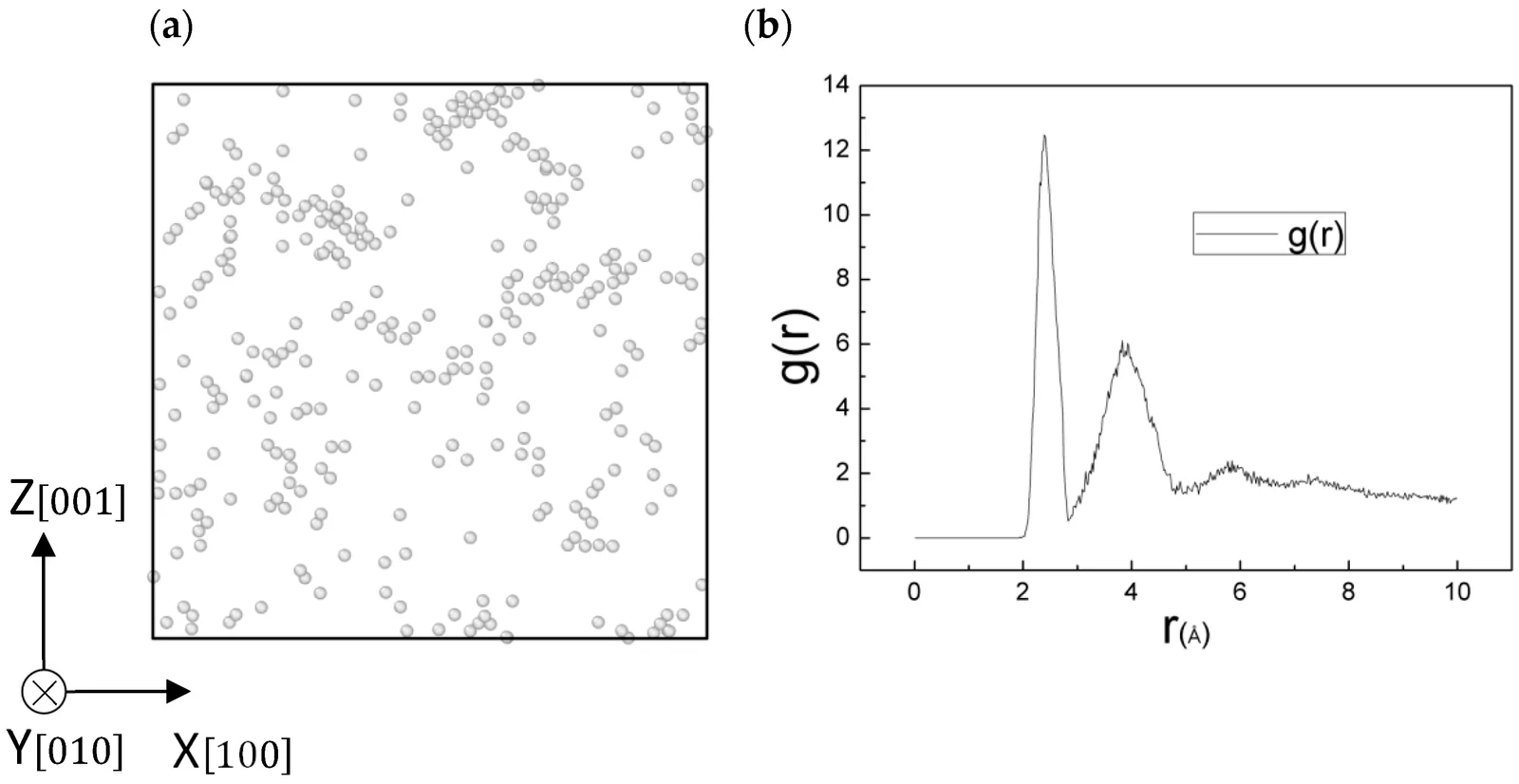
(**a**) Spatial distribution of the amorphous atoms in the system (gray spheres denote defect or amorphous atoms; crystalline atoms are omitted for clarity); (**b**) radial distribution function of the amorphous silicon. The snapshot in (**a**) and the RDF in (**b**) correspond to a compressive strain of 0.12 along the [100] direction.

Because the model is a bulk crystal with a perfect initial lattice, it contains no heterogeneous nucleation sites; the amorphous phase is therefore expected to nucleate homogeneously, which delays the onset of the crystalline-to-amorphous transition relative to crystals containing pre-existing defects. The amorphous regions nucleate homogeneously throughout the system (Figure 6a), which is characteristic of stress-assisted amorphization at high temperature. These nuclei remain spatially dispersed rather than aggregating into large clusters, consistent with homogeneous nucleation in a defect-free crystal. Figure 6b presents the radial distribution function (RDF) of the amorphous silicon formed during compression. The RDF was calculated at a compressive strain of 0.12 using only the atoms identified as amorphous at that strain, with a bin width of 0.02 Å, and was normalized by the average number density of the selected atoms; because the selected population evolves dynamically during deformation, the RDF is used here only for a qualitative characterization of short-range order. Its first peak, at about 2.4 Å, matches the Si-Si bond length of the crystalline phase at this temperature, and no additional peak appears between the first and second maxima. Both features agree with previous simulation results for amorphous silicon [28]. Only three distinct peaks are visible in the RDF, indicating that the amorphous silicon retains short-range order but lacks long-range structural order.

**Figure 7:**
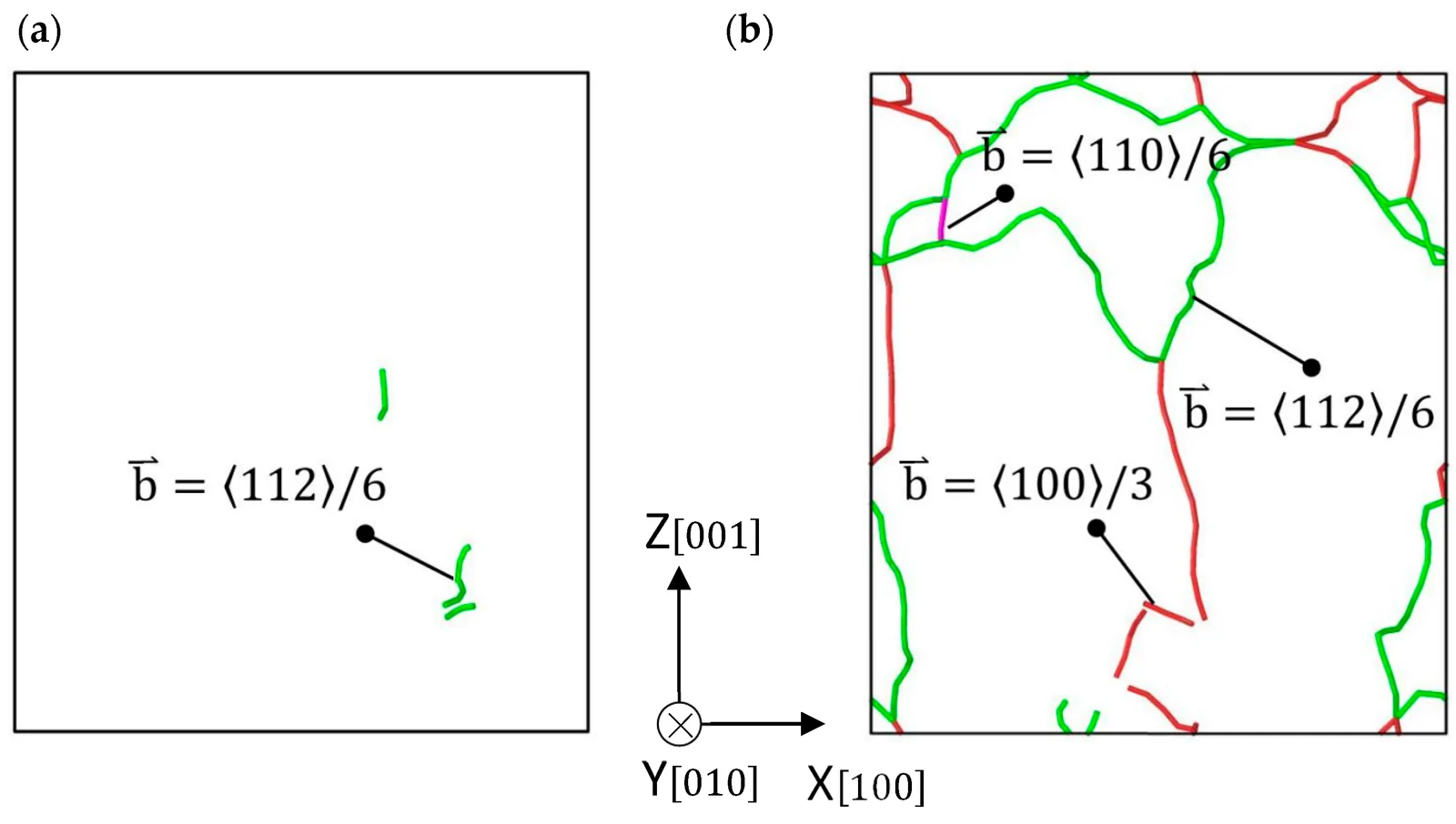
(**a**) Dislocations in the silicon crystal at the onset of plasticity; (**b**) dislocation network formed at larger strains (crystalline atoms are omitted for clarity; the green lines denote Shockley partial dislocations, the red lines full dislocations, and the magenta lines Lomer-Cottrell dislocations; the thin lines ending in dots are pointers that connect the Burgers-vector labels to the corresponding dislocation segments and convey neither the magnitude nor the direction of the Burgers vector). The snapshots correspond to compressive strains of 0.15 (**a**) and 0.20 (**b**) along the [100] direction.

Once the applied stress reaches the upper yield strength, dislocations nucleate in the silicon crystal and plastic deformation sets in, causing an abrupt stress drop, as shown in Figure 5b. Figure 7a illustrates the dislocation configuration at the onset of plasticity: only a few segments have formed, and DXA identifies them as Shockley partial dislocations with a Burgers vector of <112>/6. Their appearance causes the stress to drop to the lower yield strength (Figure 5b). At the same time, the amorphous fraction, which had accumulated uniformly before yielding, collapses as the stress relaxes; the amorphous atoms recrystallize into the diamond cubic structure. The reduced stress removes the driving force for amorphization, allowing the system to return to its energetically preferred crystalline state, and by the lower yield point, most amorphous atoms have already recrystallized, as shown in Figure 5b. After passing the lower yield point, as strain increases, dislocations continuously form and intersect with each other to form a dislocation network, as shown in Figure 7b. The dislocations formed during high-temperature plastic deformation comprise Shockley partial dislocations with a Burgers vector of <112>/6, full dislocations with a Burgers vector of <100>/3, and Lomer-Cottrell dislocations with a Burgers vector of <110>/6. This dislocation network hinders further movement of dislocations, so that continuous plastic flow requires progressively higher applied stress. Two multiplication channels can be distinguished in the subsequent deformation. On the one hand, new Shockley partials nucleate within the remaining diamond cubic regions, and existing dislocations lengthen and multiply by intersection and cross-glide on the {111} planes. On the other hand, recrystallization of the amorphous regions is itself a source of dislocations: as the disordered phase reverts to the diamond cubic structure under the reduced stress, misoriented crystallites and growth ledges form at the recrystallization front, and the associated misfit is accommodated by the emission of additional partial dislocations. The amorphous phase thus not only stores the pre-yield strain energy but also seeds part of the dislocation population that drives the subsequent plastic flow, and the interplay of these two channels produces the dislocation network shown in Figure 7b.

**Figure 8:**
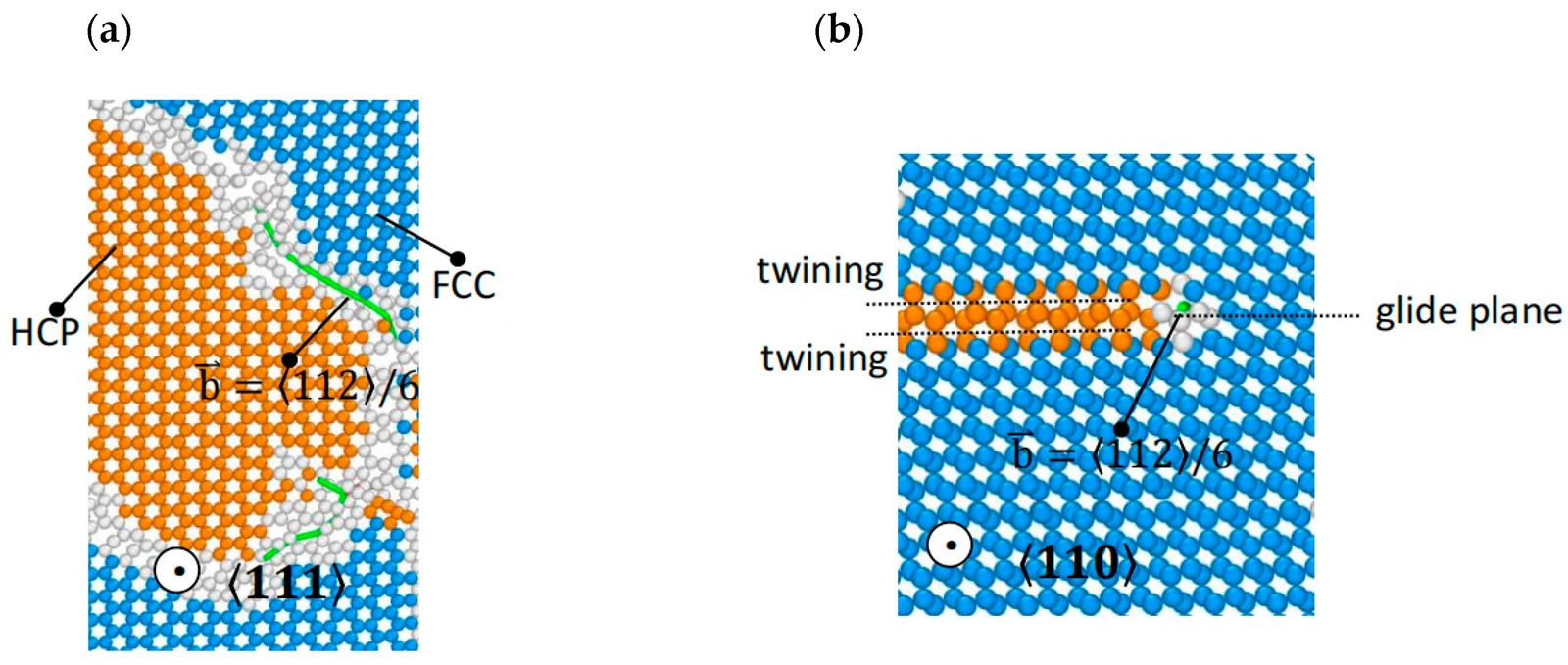
Projected local structure around the stacking fault and the Shockley partial dislocation during the strain-hardening stage: (**a**) (111) plane; (**b**) (110) plane (blue spheres denote diamond cubic atoms, orange spheres denote HCP-coordinated atoms, gray spheres denote defect or amorphous atoms, and green lines denote Shockley partial dislocations). The snapshots correspond to compressive strains of 0.20 along the [100] direction.

The generation of intrinsic stacking faults accompanies strain hardening. A stacking fault bounded by a Shockley partial dislocation loop adopts a local hexagonal-diamond arrangement consisting of two consecutive {111} bilayers; we refer to this process as stacking-fault generation rather than a phase transformation. The boundary between the stacking fault region and the surrounding host crystal is a Shockley partial dislocation whose core lies on a glide-type {111} plane, as shown in Figure 8. The high mobility of these Shockley partials on {111} planes underlies the pronounced plastic deformability of silicon at elevated temperatures [29]. These observations establish a clear sequence of mechanisms at high temperature: stress-assisted amorphization before yielding, followed by dislocation-dominated plasticity and strain hardening.

These results are consistent with and extend previous theoretical and experimental studies. Stress-induced amorphization preceding dislocation activity has been reported in MD simulations of nanocrystalline silicon under shear [18] and of silicon nanoparticles under compression [19]; in those systems, however, free surfaces and grain boundaries provided heterogeneous nucleation sites, whereas the present results demonstrate that the same sequence arises in bulk-like crystals through homogeneous nucleation. The dominance of Shockley partials gliding on {111} planes agrees with the defect energetics obtained from improved Stillinger-Weber parametrizations [28] and with the experimentally established picture of partial-dislocation-mediated plasticity above the brittle-to-ductile transition [15,16]; the strong reduction in the yield strength at T/T_m_ = 0.88 relative to low-temperature values is also consistent with the experimentally observed decrease in the yield stress of silicon as the melting point is approached [15,16]. Finally, the present simulations impose strictly uniaxial strain, whereas thermal expansion and contraction during ingot growth generate three-dimensional, multiaxial stress states. Under multiaxial loading, the hydrostatic stress component is expected to shift the critical amorphization stress and to activate additional {111} slip systems, so the critical strains reported here should be regarded as orientation-specific values; the sequential nature of the two mechanisms, which is governed by the stress level relative to the upper yield strength, is nevertheless expected to hold under more complex stress states. Extending the simulations to biaxial and triaxial loading will be an important subject of future work.

## 4. Conclusions

Molecular dynamics simulations conducted at a reduced temperature T/T_m_ = 0.88 demonstrate that the elastoplastic transition of silicon near its melting point follows a two-stage process. Under compressive loading, the diamond-cubic lattice gradually undergoes distortion; intense thermal vibrations promote partial cleavage of the Si-Si bonds, leading to uniform amorphization within the crystal. When the stress reaches the upper yield strength, Shockley partial dislocations nucleate on the {111} crystal planes, initiating plastic deformation. With stress relaxation, the driving force for amorphization is eliminated; as a result, most amorphous atoms undergo recrystallization and revert to the diamond cubic structure. As the deformation increases, dislocations multiply and interweave to form a network, accompanied by the generation of intrinsic stacking faults (locally adopting the hexagonal-diamond arrangement), indicating the characteristic work-hardening behavior. These results resolve the long-standing debate regarding whether high-temperature plasticity in silicon is governed by amorphization or dislocation activity. In fact, both mechanisms play a role, but they occur sequentially rather than competing with each other. Our findings have direct practical implications for the photovoltaic industry. During the directional solidification process, the compressive stress arising from radial thermal gradients, from volumetric expansion during solidification and contraction during cooling, and from crucible constraint can induce amorphization of the high-temperature ingot. Therefore, our results consolidate, at the atomic scale, the long-standing process guidance that controlling the thermal field and stress state near the solid–liquid interface is crucial for reducing the dislocation density in the grown multicrystalline silicon, and they identify stress-assisted amorphization and its recrystallization as the microscopic link between the thermal-mechanical conditions and dislocation multiplication. Future research should integrate atomic-scale results into continuum models to enable quantitative prediction of dislocation density in multicrystalline silicon ingots.

## Figures and Tables

**Table 1:** Young’s modulus and upper yield strength under tensile and compressive deformation at high temperature.

Direction	Tension		Compression	
	Young’s Modulus (GPa)	Yield Strength (GPa)	Young’s Modulus (GPa)	Yield Strength (GPa)
$\left[100\right]$	73.07	9.36	64.82	7.23
$\left[1\bar{1}0\right]$	119.51	8.59	104.6	7.31
$\left[111\right]$	128.95	N/A	121.09	7.18
$\left[11\bar{2}\right]$	117.19	9.14	107.17	6.52

## Data Availability

The original contributions presented in this study are included in the article. Further inquiries can be directed to the corresponding author.
